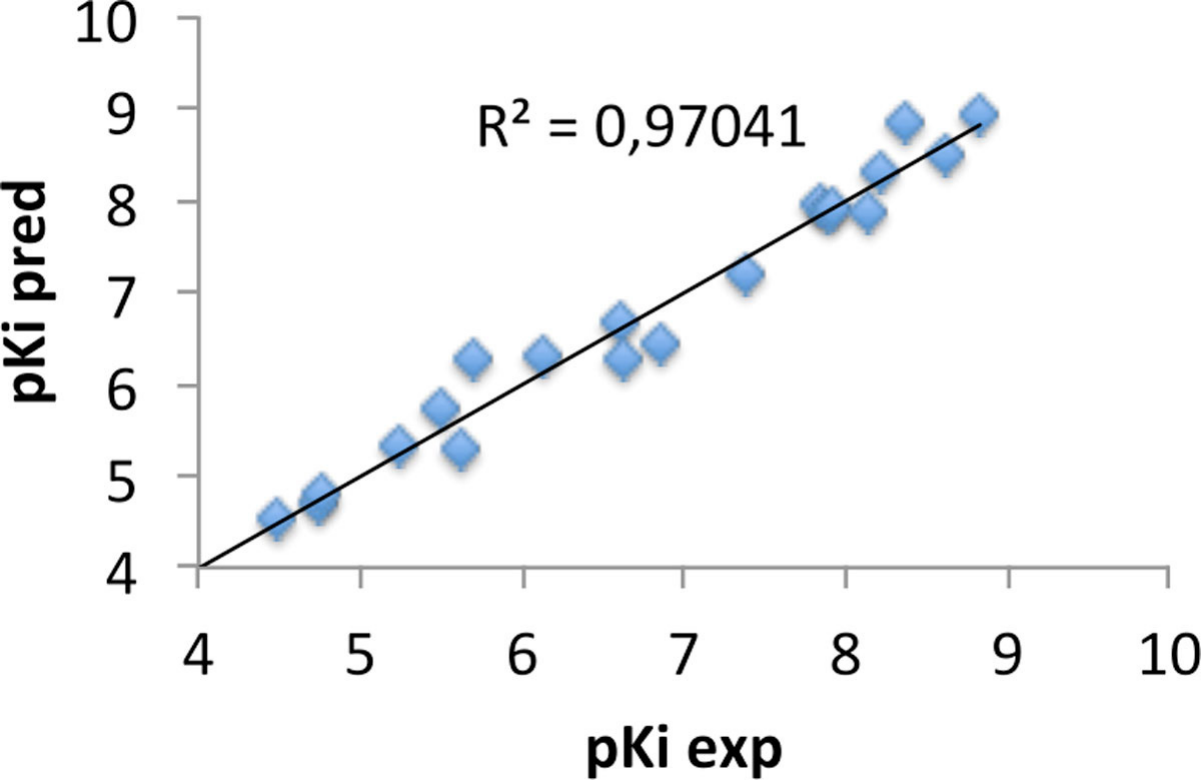# A 3D-QSAR model for cannabinoid receptor (CB2) ligands derived from aligned pharmacophors

**DOI:** 10.1186/1758-2946-5-S1-P40

**Published:** 2013-03-22

**Authors:** Robert Günther, Winnie Deuther-Conrad, Rareś Moldovan, Steffen Fischer, Peter Brust

**Affiliations:** 1 Dept. Neuroradiopharmaceuticals, Institute of Radiopharmacy, Helmholtz-Zentrum Dresden-Rossendorf, Leipzig-Site, Leipzig, 04368, Germany

## 

Cannabinoid (CB) receptors have gained much attention as markers for various brain tumours and potential therapeutic targets of neuropathic pain and mood disorders. Two CB receptors have been cloned and described: CB1, predominantly expressed in the brain and CB2, primarily found in the peripheral system but also in brain. The CB2 receptor is suggested to be involved in various neurodegenerative diseases, such as Alzheimer's or Parkinson's disease [1]. Early and non-invasive diagnosis and therapy monitoring of such diseases is desired. Positron-Emission-Tomography (PET) allows imaging of functional processes in living humans. For this, compounds with positron emitting labels like 18F are used. Due to the high sensitivity of PET, such radiotracers must bind to the target protein with high selectivity.

Here, we utilise AutoGPA [2] implemented in the modelling suite MOE (Chemical Computing Group Inc., Montreal) to compute grid potentials build upon a 3D-QSAR model derived from a library of CB2 selective N-Aryl-oxadiazolyl-propionamides. Since a proper alignment of the molecules prior the analysis is crucial to the successful application of these models in further studies, the molecules were aligned based on their pharmacophore features. The obtained model delivers also knowledge of the 3D-structure of the binding site, which, in turn, can be used to refine 3D-models of the CB2 receptor. The steric and electrostatic contour maps are applied for identification of regions suitable for labelling with 18F, the most preferred PET radionuclide.

**Figure 1:**